# The use of condoms and other birth control methods among sexually active school-going adolescents in nine sub-Saharan African countries

**DOI:** 10.1186/s12889-022-14855-6

**Published:** 2022-12-16

**Authors:** Peter Bai James, Augustus Osborne, Lawrence Sao Babawo, Abdulai Jawo Bah, Emmanuel Kamanda Margao

**Affiliations:** 1 National Centre for Naturopathic Medicine, Faculty of Health, Southern Cross University, Lismore, NSW 2480 Australia; 2 Faculty of Pharmaceutical Sciences, College of Medicine and Allied Health Sciences, University of Sierra Leone, Freetown, Sierra Leone; 3 Department of Biological Sciences, School of Environmental Sciences, Njala University, Njala, Sierra Leone; 4 Department of Nursing, School of Community Health Sciences, Njala University, Bo Campus, Bo, Sierra Leone; 5 Institute for Global Health and Development, Queen Margaret University Edinburg, Musselburgh, Scotland, UK

**Keywords:** Contraceptives, Adolescents, Sub-Saharan African, Condom use, Sexual and reproductive health

## Abstract

**Background:**

Adolescents in sub-Saharan Africa still face sexual and reproductive health challenges. Contraceptives have been used to address these challenges. Despite efforts at national and global levels, contraceptive uptake among young people in Africa remains a challenge due to personal, societal, and health systems-based barriers. We estimated the prevalence and correlates of condom use and other birth control methods among sexually active school-going adolescents in nine sub-Saharan African (SSA) countries.

**Methods:**

We conducted a secondary analysis of the Global School-based Student Health Surveys (GSHS) datasets pooled from nine SSA countries. We included a sample of 27,504 school-going adolescents 11 years and younger and 18 years and older. We employed meta-analysis using a random-effects model to estimate the total prevalence of the use of condoms, other birth control methods other than a condom and any birth control method at last sexual intercourse. We conducted complex sample descriptive and logistic regression analyses to determine the characteristics and determinants of not using condoms and other birth control methods among sexually active school-going adolescents in nine sub-Saharan African countries, respectively.

**Results:**

More than half [*n* = 4430, 53.8% (43.9–63.8)], two-fifth [*n* = 3242, 39.5% (33.2–45.9) and two-thirds of adolescents [*n* = 4838, 65.6% (57.5–73.7)] of sexually active in school adolescents across the nine sub-Saharan African countries used condom, other birth control methods and any form birth control method during their last sexual intercourse, respectively. The non-use of condoms at last sex was associated with being younger (less than 16 years) [AOR = 1.48;95%CI: 1.12–1.94], early sexual debut [AOR = 1.81(1.47–2.22)], having two or more sexual partners [AOR = 1.30(1.06–1.58)] and no/minimal parental support [AOR = 1.54(1.17–2.03)]. The non-use of other birth control methods at last sex was associated with being male [AOR = 1.37 (1.09–1.73)], early sexual debut [AOR = 1.83(1.48–2.27) and having no parental support [AOR = 1.64(1.34–2.00)].

**Conclusion:**

Contraceptive need among sexually active school adolescents in the nine sub-Saharan African countries is high. Such a need calls for the development of country-specific and or the review of existing school-based sexual health education and youth-friendly sexual and reproductive health interventions that target risky adolescents and promote adolescent-parent effective communication, connectedness and support.

**Supplementary Information:**

The online version contains supplementary material available at 10.1186/s12889-022-14855-6.

## Background

Adolescence is the period characterised by risk-taking behaviours and indulging in sexual intercourse is among risky behaviours that can lead to adverse sexual and reproductive health (SRH) outcomes. Adolescent sexual and reproductive health has become a global health concern and priority, given that their sexual and reproductive choices significantly impact their health, wellbeing, education, and global economy [1]. Early sexual debut among adolescents has been linked to an increased chance of acquiring sexually transmitted diseases, including HIV/AIDS, unintended teenage pregnancy, and negative perception of condoms [2]. In Africa, one in every four adolescents aged 15–19 years have had sex before their 15 birthday [3], and the prevalence of adolescent pregnancy in Africa is estimated at 18.8% [4]. Also, recent estimates suggest that approximately one in every three unintended pregnancies is among girls aged 15–19 in Africa [5]. A recent estimate suggests that one in four adolescent girls and close to one in five adolescent boys aged 15–19 in Eastern and Southern Africa tested positive for HIV in the past 12 months [6]. In Sub-Saharan Africa, HIV/AIDS and pregnancy-related complications are responsible for most adolescent deaths [7].

Globally, regional and national commitments and targets have been made to address adolescents’ sexual and reproductive health challenges by ensuring access to sexual and reproductive healthcare services, including information and education for adolescents [1, 8, 9]. Global health organisations such as the World Health Organisation and other United Nations organisations have championed the course of adolescent sexual and reproductive health and rights since the adoption of a resolution that recognised the need to design and implement health policies and interventions for adolescents that promote their health and wellbeing [10]. Also, instruments such as the Convention on the Elimination of All Forms of Discrimination Against Women (CEDAW) and the Universal Declaration of Human Rights recognised adolescents’ sexual and reproductive health rights [11]. The Sustainable Development Goals (SDG) also recognised adolescents’ sexual and reproductive rights. They have set a target (target 3.7) to ensure universal access to sexual and reproductive healthcare services, including family planning, information, education and integration into member states’ national health policies and programs [12]. One of the indicators to achieving this target is to reduce pregnancy rates and prevent and reduce the spread of sexually transmitted infections, including HIV/AIDS, among adolescents [12].

Achieving such a target depends on the access, consistent and effective use of modern contraceptives [13, 14]. However, studies have indicated that the use of contraceptives among adolescents in sub-Saharan Africa remains low [3, 15]. Inequality relating to gender norms, stigma and power dynamics in sexual relationships, economic and political factors such as poverty and policy support for contraceptive use, negative attitudes and beliefs about contraceptives and contraceptive use and parent-adolescent communication about SRH have been identified as barriers to adolescent contraceptive use in Africa [16]. Also, studies have reported that male sex, young age, substance abuse, mental health issues, chronic absenteeism and lack of parental and peer support have all been linked to non-use of contraceptives among adolescents [13, 14, 17–19].

Studies examining the use of condoms and other birth control methods among adolescents using a pooled dataset from different African countries are few and mostly looked at the 15 and above age bracket. For example, a study by Bankole et al. in 2007 examined correct condom use and consistency of use among adolescents(14 to 19 years) in four countries in sub-Saharan Africa and reported that consistent use of the condom was 38% in Burkina Faso, 47% in Ghana, 20% in Malawi and 36% in Uganda [20]. A recent study by Radovich and colleagues looked at the use of modern contraceptive methods among young women (age 15–24) using the most recent Demographic and Health Survey(DHS) since 2000 for 33 sub-Saharan African countries [15]. They reported that most young women reported using the short-term method of contraception, especially condoms and injections, although the use of these methods declined with each year’s increase in age [15]. Also, a study by Liang et al. examined the global contraceptive use trend among adolescents (15–19 years) using DHS data(1994–2018) and reported a modern contraceptive prevalence of 3.8% in 1994 and 14.8% in 2018 [21]. Another study by Dolyle et al. examined the sexual and reproductive behaviour of adolescents aged 15-19 years using DHS and AIDS Indicator Surveys (AIS) from 24 countries [3]. In their study, they reported that a quarter of adolescents have had sex before their 15th birthday and condom use was more prevalent among Urban youth and those with higher education [3]. Pooled data on the 10–14 years age gap is limited in Africa. Also, there is limited information regarding the general correlates of the use of condoms and other birth control methods for adolescents aged 11–18 years in Africa using data from different countries. To our knowledge, only Shayo and Kalmo have used pooled data using this age bracket [22]. However, their study assessed the prevalence and correlates of sexual intercourse among sexually active in-school adolescents in five African countries using Global School-based Student Health Surveys (GSHS) [22]. As such, this study aimed to fill this nascent gap in the current literature by examining the prevalence and correlates of the use of condoms and other birth control methods among sexually active school-going adolescents between the 11 years and younger and 18 years and older in nine sub-Saharan African countries using the 2012–2017 GSHS.

## Methods

### Study design and data source

Our study involved a secondary cross-sectional analysis of the GSHS data from nine sub-Saharan African countries (Sierra Leone, Liberia, Benin, Ghana, Mauritius, Mozambique, Namibia, Seychelles and Tanzania). GSHS is a nationally representative school-based survey that employed a two-stage cluster sample design. The first stage involves the selection of schools with probability proportional to enrollment size, while the second stage involves randomly selecting classes for which all students have equal chances of being selected [23]. It involves the use of a standardised questionnaire to collect data from school-going adolescents on ten health behaviours indicators such as alcohol use, dietary behaviours, drug use, hygiene, mental health, physical activity, sexual behaviours, tobacco use and violence and unintentional injury. A total of 27,504 school-going adolescents 11 years and younger and 18 years and older in nine sub-Saharan countries were included in our study. We adhere to the Strengthening the Reporting of Observational Studies in Epidemiology (STROBE) statement in drafting our manuscript [24].

### Study variables

Table 1 details how our outcome and independent variables were defined and measured. Our outcome variables include condom use at last sexual intercourse, other birth control methods other than condoms at last sexual intercourse and any birth control method used at last sexual intercourse. Any birth control method used at last sexual intercourse was defined as the use of either a condom or other birth control method other than a condom or both at last sexual intercourse. As shown in Table 1, the independent variables include age, sex, sexual initiation less than 14 years, current alcohol use, ever use of cannabis or amphetamine, psychological distress, school attendance, peer support, parental support and number of sexual partners. Based on previous studies [13, 18, 19], we considered no close friends, loneliness, anxiety, suicidal ideation, and suicide attempt as psychological distress items. As reported in similar studies [13, 18, 19], we summed these items into three groups – 0 = 0(no psychological distress item), 1 = 1 (single psychological distress item) and 2–5 = 2 (multiple psychological distress item). Parental supervision, parental connectedness, Parental bonding, and parental respect for privacy were considered as parental or guardian support, and they were categorised into three groups - 0–1 as low, 2 as medium and 3–4 as high support. Tobacco use was excluded in our analysis since there was a considerable number of missing data.

**Table 1:** Questionnaire items and coding scheme

Indicator	Item	Responses (coding scheme)
**Outcome Variable**		
Condom use	‘The last time you had sexual intercourse, did you or your partner use a condom?’	‘I have never had sexual intercourse, Yes, No, I do not know’
Other Birth control method use	‘The last time you had sexual intercourse, did you or your partner use any method of birth control, such as withdrawal, rhythm (safe time), birth control pills, or any other method to prevent pregnancy?	‘I have never had sexual intercourse, Yes, No, I do not know
**Independent variables**		
**Demographic variables**		
**Age**	‘How old are you?’	‘11 years old or younger to 18 years old or older’
**Sex**	**‘**What is your sex?’	‘Male, Female’
**Sexual risk**		
Number of sex partners	‘During your life, with how many people have you had sexual intercourse?’	‘I have never had sexual intercourse, 1 person to 6 or more people’
Early sexual debut	“How old were you when you had sexual intercourse for the first time mong students whoever had sexual intercourse?	‘Coded 1 = 11,12 and 13 year old and coded 2 = 14,15,16, 17 and 18 or older)’
**Substance use**		
Current alcohol use	‘During the past 30 days, on how many days did you have at least one drink containing alcohol?’	‘1 = 0 days to 7 = All 30 days (coded 1 = 0, 2–7 = 1)’
Cannabis use	‘During your life, how many times have you used marijuana	‘1 = 0 times to 5 = 20 or more times (coded 1 = 0 and 2–5 = 1)’
Amphetamine use	‘During your life, how many times have you used amphetamines or methamphetamine	‘1 = 0 times to 5 = 20 or more times (coded 1 = 0 and 2–5 = 1)’
**Psychological distress**		
No close friends	‘How many close friends do you have?’	‘1 = 0–4 = 3 or more (coded 1 + =0, 0 = 1)’
Loneliness	‘During the past 12 months, how often have you felt lonely?’	‘1 = never to 5 = always (coded 1–3 = 0 and 4–5 = 1)’
Anxiety	‘During the past 12 months, how often have you been so worried about something that you could not sleep at night?’	‘1 = never to 5 = always (coded 1–3 = 0 and 4–5 = 1)’
Suicide ideation	‘During the past 12 months, did you ever seriously consider attempting suicide?’	‘Yes, No’
Suicide attempt	‘During the past 12 months, how many times did you actually attempt suicide?’	‘1 = 0 times to 5 = 6 or more times (coded 1 = 0 and 2–5 = 1)’
**Protective factors**		
School attendance	‘During the past 30 days, on how many days did you miss classes or school without permission?’	‘1 = 0 days to 10 or more days (coded 1 = 1)’
Peer support	‘During the past 30 days, how often were most of the students in your school kind and helpful?’	‘1 = never to 5 = always (coded 1–3 = 0 and 4–5 = 1)’
Parental supervision	‘During the past 30 days, how often did your parents or guardians check to see if your homework was done?’	‘1 = never to 5 = always (coded 1–3 = 0 and 4–5 = 1)’
Parental connectedness	‘During the past 30 days, how often did your parents or guardians understand your problems and worries?’	‘1 = never to 5 = always (coded 1–3 = 0 and 4–5 = 1)’
Parental bonding	‘During the past 30 days, how often did your parents or guardians really know what you were doing with your free time?’	‘1 = never to 5 = always (coded 1–3 = 0 and 4–5 = 1)’
Parental respect for privacy	‘During the past 30 days, how often did your parents or guardians go through your things without your approval?’	‘1 = never to 5 = always (coded 1–3 = 0 and 4–5 = 1)’

### Ethical consideration

We did not seek ethics approval, given that our study was based on a secondary analysis of publicly available data. However, ethics approval was sought from the ministries of health in the respective countries prior to collecting the primary data.

### Statistical analysis

We used SPSS version 28 to analyse our pooled data. We presented categorical variables using unweighted frequencies and weighted percentages. Given the significant heterogeneity between countries (I^2^ > 95%), we employed meta-analysis using a random-effects model to estimate the total prevalence of condom use, other birth control methods other than condoms and any birth control method used at last sex intercourse. We employed bivariate (chi-square test) and multivariate binary logistic regression analysis to determine the determinants associated with condom use, other birth control methods other than a condom and the use of any birth control method at last sexual intercourse. In both bivariate (chi-square test) and multivariate binary logistic regression analysis, we used a complex sampling command on SPSS to account for sample weights and sampling design effect. Model fitness was tested using the Hosmer–Lemeshow test, which shows that our models were fit for condom use (*p* = 0.832), other birth control methods other than a condom (*p* = 0.812) and any birth control use (*p* = 0.823). We used the variance inflation factor (VIF) to test for multicollinearity, and no evidence of multicollinearity was observed among the independent variables (VIF < 1.5) (See additional file 1).

## Results

The sample consisted of 27,504 adolescents in nine sub-Saharan African countries, and there were slightly more males[*n* = 13,444,52.7%] than females [*n* = 13,632, 47.7%)]. Close to two-thirds were below the age of 16 [*n* = 13,776, 59.3%], and approximately two out of every five [*n* = 2977, 42.6%] had sex before their 14th birthday (See Table 3 for details).

Table 2 shows the prevalence of condom use and other birth control methods other than a condom and any birth control method used at their last sexual encounter among school-going adolescents in nine sub-Saharan African countries. We found that approximately two-fifth of school-going adolescents in our study [*n*=,9463, 42.6% (31.9–53.2)] had ever had sex. Liberia had the highest prevalence of ever-had sex, [*n* = 1264, 61.9% (60.1–63.7)] while Tanzania had the lowest prevalence of ever-had sex, [*n* = 668, 20.0% (18.7–21.3)]. More than half of sexually active adolescents in the nine sub-Saharan African countries used a condom during their last sexual intercourse [*n* = 4430, 53.8% (43.9–63.8)]. Namibia had the highest prevalence of condom use at last sexual intercourse [*n*=,1272, 75.8% (74.6–77.0)], while Tanzania had the lowest prevalence of condom use at last sexual intercourse, [*n* = 128, 36.5% (35.0–38.0)]. More than one-third of sexually active school-going adolescents used other birth control methods other than condoms at their last sexual intercourse [*n* = 3242, 39.5% (33.2–45.9), with Liberia having the highest prevalence [*n* = 581, 53.4(51.5–55.3)]. Approximately two-thirds of adolescents who have ever had sex were found to have used any form of birth control method during their last sexual intercourse [*n* = 4838, 65.6%(57.5–73.7)] with Namibia[*n* = 1317, 82.0% (80.9–83.1)] and Tanzania [*n* = 143, 47.0%(45.4–48.6)] reporting the highest and lowest prevalence respectively.

**Table 2:** Prevalence of condom use, other birth control method use other than condom and any birth control method use at last sex among school-going adolescents in nine sub-Saharan African Countries using 2012–2017 GSHS

Country	Year	Ever had sex		Condom use at last sexual intercourse		Other birth control method other than condom at last sex intercourse		Any birth control method use at last sexual intercourse	
		Unweighted count	% (95%CI)	Unweighted count	% (95%CI)	Unweighted count	% (95%CI)	Unweighted count	% (95%CI)
Total(yes)		9463	42.6 (31.9–53.2)	4430	53.8 (43.9–63.8)	3242	39.5 (33.2–45.9)	4838	65.60 (57.5–73.7)
Sierra Leone(yes)	2017	917	38.9(37.1–40.7)	325	41.0(39.2–42.8)	376	49.3 (47.4–51.2)	430	64.0 (62.2–65.8)
Liberia (yes)	2017	1264	61.9 (60.1–63.7)	632	60.2 (58.4–62.0)	581	53.4 (51.5–55.3)	691	75.3 (73.7–76.9)
Benin(yes)	2016	1133	53.4 (51.5–55.3)	512	48.8 (46.9–50.7)	388	36.0 (34.1–37.9)	595	57.4 (55.3–59.3)
Ghana(yes)	2012	1117	36.9 (35.3–38.5)	402	47.6 (46.0–49.2)	367	40.8 (39.2–42.4)	478	62.3 (60.7–63.9)
Mauritius (yes)	2017	517	20.6 (19.2–22.0)	190	49.1 (47.3–50.9)	106	25.8 (24.2–27.4)	221	62.1 (60.4–63.8)
Mozambique(yes)	2015	993	57.4 (55.2–59.6)	596	75.0 (73.1–76.9)	374	48.5 (46.3–50.7)	551	79.4 (77.6–81.2)
Namibia(yes)	2013	2021	53.7 (52.2–55.2)	1272	75.8 (74.6–77.0)	733	42.9 (41.5–44.3)	1317	82.0 (80.9–83.1)
Seychelles(yes)	2015	833	40.4 (38.5–42.3)	373	50.5 (48.6–52.4)	197	26.1 (24.4–27.8)	412	60.8 (58.9–62.7)
Tanzania(yes)	2014	668	20.0 (18.7–21.3)	128	36.5 (35.0–38.0)	120	33.1 (31.6–34.6)	143	47.0 (45.4–48.6)

Table 3 summarises the characteristics of the birth control method used among school-going adolescents in nine sub-Saharan African countries. From our study, approximately one in four sexually active school-going adolescents (*n* = 1132, 27.6%) aged 16 or less used condoms during their last sexual encounter. Regarding gender differences, more males (*n* = 2594, 62.9%) than females (*n* = 1776, 37.1%) used a condom during the last sexual encounter. A similar pattern was observed regarding using other birth control methods other than condoms and any birth control method. Close to half (*n* = 2227, 47.7%) of sexually active school-going adolescents who had two or more sexual partners used a condom during their last sexual encounter. We observed a similar pattern regarding using other birth control methods besides condoms. Close to one-third of sexually active adolescents who had sex before the age of 14 years used condoms (*n* = 1187, 30.8), while approximately two in five sexually active school-going adolescents with little or no parental support were using a condom (*n* = 1986, 44.5%) compared to those who had parental support (*n* = 1023, 28.0%). A similar pattern was observed regarding using other birth control methods other than condoms and any birth control method. Although not statistically significantly less than one-fourth of sexually active school-going adolescents who show more signs of psychological distress(*n* = 1053,23.4%) had used condoms in their last sexual encounter, whereas close to half (*n* = 1960, 48.6%) of those who show no signs of psychological distress had used condoms in their last sexual encounter.

**Table 3:** Birth control method use characteristics among school-going adolescents in nine sub-Saharan African Countries using 2012–2017 GSHS

Study characteristics		Sample	Condom use at last sexual intercourse (Yes)		Other birth control method other than condom at last sex Intercourse (Yes)		Any birth control method use at last sexual intercourse (Yes)	
	Variables	Total ***n*** (%^**a**^)	***n*** (%^**a**^)	***p***-value	***n*** (%^**a**^)	***p***-value	***n*** (%^**a**^)	***p***-value
Age group	Less than 16 years	13,776 (59.3)	1132 (27.6)	< 0.001	764 (27.5)	< 0.001	1163 (24.2)	< 0.001
	16 years and above	13,500 (40.7)	3251 (72.4)		2438 (72.5)		3626 (75.8)	
Age of sexual initiation less than 14 years	Yes	2977 (42.6)	1187 (30.8)	< 0.001	821 (31.0)	< 0.001	1286 (29.0)	< 0.001
	No	4844 (57.4)	2813 (69.2)		2068 (69.0)		3174 (71.0)	
Sex	Male	13,444 (52.3)	2594 (62.9)	0.010	1836 (60.8)	< 0.001	2830 (63.0)	< 0.001
	Female	13,632 (47.7)	1776 (37.1)		1349 (39.2)		1942 (37.0)	
Current alcohol use	Yes	6122 (13.4)	1607 (28.7)	0.561	1088 (27.6)	0.337	1780 (28.7)	0.788
	No	19,604 (86.6)	2530 (71.3)		1918 (72.4)		2749 (71.3)	
Ever use cannabis/ amphetamine	Yes	1858 (6.2)	476 (10.5)	0.838	387 (11.4)	0.998	523 (9.6)	0.275
	No	22,000 (93.8)	3394(89.5)		2403 (88.6)		3704 (90.4)	
Psychological distress items	0	13,942 (58.7)	1960 (48.6)	0.568	1337 (47.7)	0.187	2180 (50.3)	0.171
	1	6415 (24.0)	1091 (28.0)		805 (27.3)		1184 (26.6)	
	2–5	5094 (17.3)	1053 (23.4)		841 (25.0)		1151 (23.1)	
Missed class/school	Yes	8099 (31.1)	1596 (38.2)	0.284	1237(39.9)	0.990	1772 (37.8)	0.311
	No	18,640 (68.9)	2732 (61.8)		1938 (60.1)		2975 (62.2)	
Peer support	Yes	8343 (32.3)	1333 (31.5)	0.013	998 (30.0)	0.458	1463 (32.0)	0.011
	No	18,348 (67.7)	2996 (68.5)		2173(70.0)		3277 (68.0)	
Parental support	Low	11,193 (41.2)	1986 (44.5)	0.004	1420 (43.3)	< 0.001	2198 (42.9)	< 0.001
	Medium	6949 (27.0)	1140 (27.5)		841 (27.7)		1240 (28.0)	
	High	7513 (31.8)	1023 (28.0)		782 (29.0)		1134 (29.1)	
**Two or more sexual partners**	Yes		2227 (47.7)	0.001	1606 (49.7)	0.235	2521 (52.4)	0.323
	No		2149 (52.3)		1579 (50.3)		2263 (47.6)	

*n* Unweighted Count

^a^weighted percentage

Table 4 summarises the determinants of no use of condom use, no use of other birth control methods other than a condom and no use of any birth control method used at last sex among sexually active school-going adolescents in nine sub-Saharan African countries. We observed that males were more likely than females not to have used other birth control methods other than a condom [AOR = 1.37;95%CI: 1.09–1.73] and any birth control method [AOR = 1.48;95%CI: 1.22–1.80] in their last sexual encounter. Sub-analysis based on age indicated that males were more likely than females not to have used condoms among those who were 16 years and above [AOR = 1.32;95%CI:1.02–1.72]. This pattern was observed regarding the use of other birth control methods other than condoms or any birth control in general (See additional file 2 Table B). Those who have had sex before their 14th birthday were more likely not to have used condoms [AOR = 1.81;95%CI:1.47–2.22], other birth control methods [AOR = 183;95%CI:148–2.27) or any form of birth control [AOR = 2.29;95%CI:183–2.97). Also, sub-analysis based on sex and age did not show any variation (See additional file 2 tables A&B).

**Table 4:** Determinants of not using condoms, other birth control methods other than condom and any birth control method at last sex among school-going adolescents in nine sub-Saharan African Countries using 2012–2017 GSHS

Study characteristics	Not using Condom at last sex	Not using of other birth control method at last sex AOR (95%CI)	Not using of any form birth control method AOR (95%CI)
**Age group**:			
16 years and above	1	1	1
Less than 16 years	1.48 (1.13–1.95) ^⁎⁎^	1.19 (0.91–1.56)	1.55 (1.15–2.10) ^⁎⁎^
**Sex**			
(male vs female)	1.26 (0.98–1.60)	1.37 (1.09–1.73) ^⁎⁎^	1.48 (1.22–1.80) ^⁎⁎^
**Age of sexual initiation less than 14**			
(yes vs no)	1.81 (1.47–2.22) ^⁎⁎^	1.83 (1.48–2.27) ^⁎⁎^	2.29 (1.83–2.97) ^⁎⁎^
**Current alcohol use**			
(yes vs no)	0.86 (0.72. -1.04)	1.11 (0.92–1.34)	0.95 (0.80–1.14)
**Ever use cannabis/ amphetamine**			
(Yes vs No)	1.00 (0.73–1.37)	0.67 (.0.49–0.92)	0.83 (0.60–1.14)
**Two or more sexual partners**			
(yes vs. no)	1.30 (1.06–1.58) ^⁎⁎^	0.96 (0.80–1.17)	1.04 (0.86–1.24)
**Psychological distress items**:			
0	1.00 (0.77–1.30)	1.14 (0.90–1.44)	1.00 (0.75–1.33)
1	1.10 (0.82–1.46)	1.25 (0.94–1.65)	1.27 (0.94–1.72)
2–5	1	1	1
**School attendance**			
(no vs. yes)	0.89 (0.73–1.09)	1.00 (0.83–1.19)	0.92 (0.77–1.09)
**Peer support**			
(No vs Yes)	1.16 (0.96.- 1.40)	1.08 (0.88–1.32)	1.04 (0.85–1.28)
**Parental support**:			
Low	1.54 (1.17–2.03) ^⁎⁎^	1.64 (1.34–2.00) ^⁎⁎^	2.04 (1.54–2.69) ^⁎⁎^
Medium	1.10 (0.82–1.46)	1.17 (0.93–1.47)	1.22 (0.88–1.69)
High	1		1

⁎⁎ = *p* > 0.001

Regarding age, we found that those under 16 years were more likely not to use condoms [AOR = 1.48;95%CI: 1.12–1.94] and any birth control method [AOR = 1.55;95%CI: 1.15–2.10]. A similar pattern was observed between males and females (See additional file 2 Table A). Sexually active school-going adolescents with two or more sexual partners were more likely not to have used condoms during their last sexual intercourse than those without two or more sexual partners [AOR = 1.30;95%CI:1.06–1.58]. and this was observed among females [AOR = 1.66;95%CI:1.16–2.39) but not males [AOR = 11.16;95%CI:1.0.93–1.44] (See additional file 2 Table A). However, no significant association was observed regarding the use of other birth control methods other than a condom [AOR = 0.96;95%CI:0.80–1.17] and any birth control method [AOR = 1.04;95%CI:0.86–1.24]. Similar associations were observed based on sex and age. (See additional file 2 tables A&B).

Regarding protective factors, we found that sexually active school-going adolescents who had no parental support were more likely not to have used condoms [AOR = 1.54;95%CI:1.17–2.03] other birth control methods other than a condom [AOR = 1.64;95%CI:1.34–1.200] and any birth control method [AOR = 2.04;95%CI:1.54–2.69] at their last sexual encounter. Sub-analysis based on sex revealed that the association between no parental support and not using condoms, other birth control methods other than a condom and any birth control was observed among males but not females (See additional file 2 Table A). On the other hand, sub-analysis based on age shows that the association between no parental support and not using condoms was only observed among those 16 years and above [AOR = 1.52;95%CI:1.13–2.04). In comparison, there was no age difference regarding the association between no parental support and not using other birth control methods other than a condom and any birth control(See additional file 2 Table B).

## Discussion

In this study, we determined the prevalence of condom use, other birth control methods other than a condom and any birth control method at last sex among sexually active school-going adolescents in nine sub-Saharan African countries. In our study, approximately two-thirds of sexually active school-going adolescents have used one form of contraception during their last sexual encounter, which is higher than a similar study conducted in four Caribbean countries [19] but lower than the prevalence reported in a study conducted in Europe, Israel, and Canada [25].

Although condom use is known to prevent pregnancy and reduces the risk of being infected with HIV/AIDS and sexually transmitted diseases, approximately half of sexually active adolescents in the nine sub-Saharan African countries used a condom during their last sexual intercourse. Our finding is in line with a survey conducted among adolescents in four Southeast Asian countries [26], South African youths aged 15 to 24 years and out-of-school young people in Uganda [27, 28]. On the other hand, our finding is higher than the prevalence of condom use among school-going adolescents in four Caribbean countries [19]. We observed that only two out of five sexually active school-going adolescents in the nine countries use birth control methods other than condoms during their last sexual encounter, which is consistent with what was recently reported among school-going adolescents in the Caribbean [19].

Consistent with previous studies [29, 30], the low uptake of other birth controls methods other than condoms may be attributed to limited access to modern contraceptive methods such as birth control pills, IUD or implant; or a shot, patch, or birth control ring among adolescents due to relatively high cost and availability. Also, low contraceptive literacy, perceived side effects, and lack of perceived risk of sexually transmitted infection (STIs) may also serve as barriers [31–33]. Discussion around contraception and sexuality within African families is still a taboo [34, 35]. At the same time, gender norms that allow boys/men to make decisions regarding sex and contraceptive use have been identified as barriers and may help explain the relatively low uptake of birth control methods in this study. Another factor is the stigma associated with using condoms among adolescents, boys and girls. Studies have reported widespread myths that girls who use a condom are promiscuous, untrustworthy, and likely to be infected with sexually transmitted infections and that real men do not use condoms [36, 37].

Interestingly, the lowest condom and other contraceptive use prevalence were observed among school-going adolescents in Tanzania. Previous Tanzania studies have reported low uptake of condoms among female sex workers and low acceptability of condom promotion and distribution among adolescents [38–41]. Reasons such as condoms promoting promiscuity and improper use of condoms are a sin against God and can cause sexually transmitted diseases have been put forward to explain the low uptake of condoms by adolescents in Tanzania [39]. Consistent with previous studies [13, 14], the highest prevalence of condom use was observed among school-going adolescents in Mozambique and Namibia. The high use of contraceptives in these countries may be attributed to increased sexual reproductive health education and promotion in response to the high prevalence of sexual risk behaviour among adolescents reported in these countries [42–44].

In line with other studies [14, 17–19, 41, 45], our multivariate analysis indicates that male and younger adolescents were likelier not to use condoms and other birth control methods. The gender disparity regarding condom use in our study may be related to gender norms regarding sexuality in most African societies. Females are often not expected to become pregnant until marriage; therefore, those who are sexually active often tend to prevent themselves from being pregnant [46]. Also, gender differences regarding the barriers to using a condom may help explain our findings. It has been reported that males experience more barriers to condom use than women, including the perception that condoms promote negative sexual experiences [47, 48]. In addition, condomless sex has been attributed to the concept of manhood and masculinity and is a symbol of prestige among their male peers [49].

With regard to age, older adolescents are much more aware of their sexuality, have been exposed to sexual and reproductive health education to know about the consequences of unprotected sex, and can make informed decisions regarding the use of condoms and other birth control methods compared to their younger peers. Early sexual debut was associated with no use of condoms in our study, and our finding corroborates with similar national studies conducted elsewhere [2, 50]. Early sexual debut has been linked to unintended adolescent pregnancy, STIs and high-risk behaviours in adult life [2, 50].

We also observed that those with two or more sexual partners were more likely not to use condoms compared to those with a single sexual partner. Our finding is supported by previous studies [45, 51], although in contrast with a community-based study in four districts in Tanzania [41]. Adolescents in a monogamous relationship are more likely to be aware of the perceived risk associated with indulging in unprotected sex might explain our finding. Also, adolescents with multiple sexual partners are more likely to practice other at-risk sexual behaviours, such as smoking and binge drinking [52]. Condomless sex among adolescents with multiple sexual partners is a public health risk as it promotes the spread of STIs, including HIV/AIDS. We also observed that parental support was a protective factor as it promotes the use of condoms and other birth control methods. A similar association has been reported in previous studies examining the association between parental support and young people’s sexual behaviour [14, 17, 19, 53, 54]. Parental support can lead to adolescents feeling loved and, therefore, may want to live up to their parent’s expectations and likely not indulge in risky sexual behaviour that would lead to pregnancy or STI. Another possible reason for our finding is that adolescents may fear being punished by their parents if their risky sexual behaviour leads to pregnancy or STI [55]. Further, parental support has been shown to enhance adolescents’ social skills and help reduce peer influence on adolescents’ sexual decision-making process [56].

### Policy and practice implications and future research

Our findings suggest that adolescent reproductive health needs are largely unmet in these African countries despite recent progress that has been made over the years. The consequences of such an unmet need are increased adolescent pregnancy and STI infections, early marriage, school drop-out, and maternal and neonatal morbidity and mortality, which promote existing poverty. Our findings suggest the need for school-based sexual health education programs since they have been shown to have the potential to promote contraceptive use among adolescents in Sub-Saharan Africa [57]. This includes integrating sexual and reproductive health education into existing school curricula, training teachers and peer educators, and using youth activists and celebrities as sexual and reproductive health ambassadors. However, the school-based interventions should employ a combination of faith- and culture, public health and rights-based approaches depending on the context to ensure it is acceptable and achieve the desired outcome. Also, outside the school environment, providing youth-friendly sexual and reproductive health services is another avenue to address the SRH needs of adolescents [58]. This includes training and educating stakeholders, engaging adolescents to improve SRH knowledge via media, community events and use of peer-support workers and providing support to clinicians such as recruiting peer navigators that work alongside clinicians to enhance referral and linkage to youth-friendly sexual and reproductive health services [59].

Male, younger adolescents, early sexual debut, and those with two or more sexual partners were less likely to use a condom or other birth control methods, suggesting that adolescents with such characteristics are a risk group and that school-based or youth-friendly interventions should consider them as potential targets. Also, parental support was identified as a protective factor in our study, which suggest the need to develop interventions or incorporate parents into existing adolescent sexual and reproductive program to promote adolescent-parent communication, connectedness and support. In doing so, parents need to be trained to improve their knowledge and capacity to engage in conversations regarding their child’s sexual health issues and develop strategies to address socio-cultural and religious barriers that prevent effective parental engagement with their child [60].

### Study strengths and limitations

A key strength of our study is that it uses nationally representative samples of high school students in nine sub-Saharan countries, and the use of meta-analysis to account for heterogeneity to determine the overall prevalence of condoms and other birth control methods use strengthens the robustness of our methodology and validity of our findings. Notwithstanding, our study has some limitations that need to be considered when interpreting our findings. First, our study only targets school-going adolescents in these nine countries, and these may not be representative of the entire adolescent population in these countries. Future studies should look at out-of-school adolescents, especially vulnerable sub-population such as those living in informal settlements. Second, data collected were based on self-reported adolescents’ sexual activity, which increases the tendency to over or under-report their sexual and reproductive behaviour. Third, the study employed a cross-sectional design, and causality cannot be inferred. Fourth, we excluded tobacco use as a potential explanatory variable since a large amount of data on this variable were missing in the publicly available file.

## Conclusion

Our study suggests that close to half, approximately half, and one-third of sexually active school-going adolescents in the nine African countries do not use a condom, other birth control methods other than a condom and any form of birth control method, respectively, during the last sexual intercourse. Also, being a male, young, early sexual debut, those with two or more sexual partners and no parental support were associated with no use of condoms and other birth control methods. Our findings underscore the need for school-based sexual health education and youth-friendly sexual and reproductive health interventions that target at-risk adolescents and promote effective communication, connectedness, and support for adolescent-parent.

## Supplementary Information

**Additional file 1.** Collinearity Statistics for Condom use. Collinearity Statistics for the use of other birth control method. Collinearity Statistics for the use of any birth control method.

**Additional file 2: Table A.** Determinants of not using condoms, other birth control methods other than condom and any birth control method at last sex among school-going adolescents in nine sub-Saharan African Countries using 2012–2017 GSHS based on sex. **Table B.** Determinants of not using condoms, other birth control methods other than condom and any birth control method at last sex among school-going adolescents in nine sub-Saharan African Countries using 2012–2017 GSHS based on age.

## Data Availability

The datasets informing the findings of this study are publicly available. It can be freely available via the WHO NCD Microdata Repository. https://extranet.who.int/ncdsmicrodata/index.php/catalog/GSHS.

## Abbreviations

CI: Confident Intervals
VIF: Variance Inflation Factor
SRH: Sexual and Reproductive health
GSHS: Global School Health Survey
AOR: Adjusted odds ratio
STI: Sexual transmitted diseases
SPSS: Statistical Package for Social Sciences
WHO: World Health Organisation
SDG: Sustainable Development goals
CEDAW: Elimination of All Forms of Discrimination Against Women
