# Supplementary material for: The use of condoms and other birth control methods among sexually active school-going adolescents in nine sub-Saharan African countries
Source: BMC Public Health. 2022 Dec 16;22:2358. doi: 10.1186/s12889-022-14855-6 (PMC9756616; doi:10.1186/s12889-022-14855-6)
Supplement: Supplementary file 1 — Additional file 1. Collinearity Statistics for Condom use. Collinearity Statistics for the use of other birth control method. Collinearity Statistics for the use of any birth control method. [file 12889_2022_14855_MOESM1_ESM.docx]

**Collinearity Statistics for Condom use**

| Explanatory variables | Tolerance | | VIF |  |
| --- | --- | --- | --- | --- |
| Sex | .915 | 1.093 | | |
| Age of sexual initiation <14 years | .762 | 1.312 | | |
| 2 or more sex partners | .913 | 1.095 | | |
| Current alcohol use | .910 | 1.098 | | |
| School attendance | .947 | 1.056 | | |
| Peer support | .941 | 1.063 | | |
| Ever Cannabis and Amphetamine use | .919 | 1.088 | | |
| Psychological distress | .974 | 1.027 | | |
| Parental support | .918 | 1.089 | | |
| Age group | .794 | 1.259 | | |

**Collinearity Statistics for the use of other birth control method**

| Explanatory variables | Tolerance | | VIF |
| --- | --- | --- | --- |
| Sex | .913 | 1.095 | |
| Age of sexual initiation <14 years | .767 | 1.304 | |
| 2 or more sex partners | .917 | 1.090 | |
| Current alcohol use | .906 | 1.104 | |
| School attendance | .943 | 1.061 | |
| Peer support | .941 | 1.062 | |
| Ever Cannabis and Amphetamine use | .911 | 1.098 | |
| Psychological distress | .970 | 1.031 | |
| Parental support | .916 | 1.091 | |
| Age group | .801 | 1.249 | |

**Collinearity Statistics for the use of any birth control method**

| Explanatory variables | Tolerance | | VIF |
| --- | --- | --- | --- |
| Sex | .913 | 1.096 | |
| Age of sexual initiation <14 years | .763 | 1.311 | |
| 2 or more sex partners | .912 | 1.097 | |
| Current alcohol use | .907 | 1.103 | |
| School attendance | .945 | 1.058 | |
| Peer support | .942 | 1.062 | |
| Ever Cannabis and Amphetamine use | .917 | 1.090 | |
| Psychological distress | .974 | 1.027 | |
| Parental support | .918 | 1.089 | |
| Age group | .797 | 1.255 | |
