# Supplementary material for: The use of condoms and other birth control methods among sexually active school-going adolescents in nine sub-Saharan African countries
Source: BMC Public Health. 2022 Dec 16;22:2358. doi: 10.1186/s12889-022-14855-6 (PMC9756616; doi:10.1186/s12889-022-14855-6)
Supplement: Supplementary file 2 — Additional file 2: Table A. Determinants of not using condoms, other birth control methods other than condom and any birth control method at last sex among school-going adolescents in nine sub-Saharan African Countries using 2012–2017 GSHS based on sex. Table B. Determinants of not using condoms, other birth control methods other than condom and any birth control method at last sex among school-going adolescents in nine sub-Saharan African Countries using 2012–2017 GSHS based on age. [file 12889_2022_14855_MOESM2_ESM.docx]

**Table A: Determinants of not using condoms, other birth control methods other than condom and any birth control method at last sex among school-going adolescents in nine sub-Saharan African Countries using 2012-2017 GSHS based on sex**

| **Study characteristics** | **Not using Condom at last sex** | | **Not using of other birth control method** **at last sex**  **AOR (95%CI)** | | **Not using of any form birth control method**  **AOR (95%CI)** | |
| --- | --- | --- | --- | --- | --- | --- |
|  | **Male AOR (95%CI)** | **Female** **AOR (95%CI)** | **Male AOR (95%CI)** | **Female** **AOR (95%CI)** | **Male AOR (95%CI)** | **Female** **AOR (95%CI)** |
| **Age group**: 16 years and above  Less than 16 years | 1 | 1 | 1 | 1 | 1 | 1 |
|  | **1.41(1.06-1.87)** | **1.84(1.20-2.81)** | 1.13(0.82-1.56) | 1.35(0.86-2.10) | **1.42(1.07-1.88)** | **1.99(1.08-3.68)** |
| **Age of sexual initiation less than 14** (yes vs no) | **1.89(1.50-2.37)** | **1.88(1.22-2.88)** | **1.75(1.34-2.28)** | **2.09(1.35-3.25)** | **2.41(1.87-3.11)** | **2.16(1.32-3.52)** |
| **Current alcohol use** (yes vs no) | 0.85(0.67-1.09) | 0.81(0.59-1.11) | 1.03(0.83-1.27) | 1.30(0.99-1.69) | 0.88(0.72-1.08) | 1.09(0.76-1.56) |
| **Ever use cannabis/ amphetamine** (Yes vs No) | 1.28(0.87-1.90) | 0.53(0.25-1.10) | **0.63(0.44-0.93)** | 0.71(0.40-1.25) | 0.86(0.58-1.27) | 0.75(0.39-1.45) |
| **Two or more sexual partners** (yes vs. no) | 1.16(0.93-1.44) | **1.66(1.16-2.39**) | 0.90(0.71-1.14) | 1.05(0.79-1.39) | 1.01(0.79-1.29) | 1.07(0.79-1.44) |
| **Psychological distress items**: 0 1  2-5 | 0.90(0.66-1.22) | 1.30(0.84-2.00) | 1.25(0.89-1.74) | 0.99(0.71-1.37) | 0.94(0.66-1.35) | 1.13(0.71-1.82) |
|  | 1.07(0.77-1.48) | 1.26(0.78-2.04) | 1.45(0.99-2.12) | 1.01(0.68-1.50) | 1.33(0.93-1.91) | 1.17(0.68-2.01) |
|  | 1 | 1 | 1 | 1 | 1 | 1 |
| **School attendance** (no vs. yes) | 0.80(0.65-1.00) | 1.07(0.74-1.54) | 1.03(0.83-1.28) | 0.91(0.64-1.30) | 0.84(0.69-1.02) | 1.00(0.72-1.39) |
| **Peer support** (No vs Yes) | 1.02(0.80-1.30) | **1.58(1.21-2.07)** | 1.16(0.91-1.48) | 0.89(0.64-1.24) | 1.05(0.80-1.38) | 1.18(0.86-1.63) |
| **Parental support**: Low  Medium  High | **1.89(1.35-2.64)** | 1.03(0.67-1.58) | **2.02(1.54-2.65)** | 1.07(0.77-1.49) | **2.35(1.67-3.30)** | 1.44(0.90-2.30) |
|  | 1.10(0.78-1.56) | 1.10(0.68-1.78) | **1.34(1.01-1.79)** | 0.85(0.56-1.28) | 1.22(0.84-1.78) | 1.17(0.65-2.12) |
|  | 1 | 1 | 1 | 1 | 1 | 1 |

**Table B: Determinants of not using condoms, other birth control methods other than condom and any birth control method at last sex among school-going adolescents in nine sub-Saharan African Countries using 2012-2017 GSHS based on age**

| **Study characteristics** | **Not using Condom at last sex** | | **Not using of other birth control method** **at last sex**  **AOR (95%CI)** | | **Not using of any form birth control method**  **AOR (95%CI)** | |
| --- | --- | --- | --- | --- | --- | --- |
|  | **Less than 16 years AOR (95%CI)** | **16 years and above AOR (95%CI)** | **Less than 16 years AOR (95%CI)** | **16 years and above AOR (95%CI)** | **Less than 16 years AOR (95%CI)** | **16 years and above AOR (95%CI)** |
| **Sex** (male vs female) | 1.09(0.71-1.66) | **1.32(1.02-1.72)** | 0.98(0.58-1.65) | **1.45(1.14-1.86)** | 1.08(0.64-1.81) | **1.61(1.27-2.04)** |
| **Age of sexual initiation less than 14** (yes vs no) | **2.02(1.31-3.11)** | **1.75(1.41-2.16)** | **2.50(1.62-3.86)** | **1.70(1.35-2.15)** | **2.81(1.75-4.49)** | **2.22(1.75-2.81)** |
| **Current alcohol use** (yes vs no) | 1.29(0.85-1.95) | **0.77(0.61-0.98)** | 1.14(0.69-1.87) | 1.11(0.90-1.37) | 1.10(0.65-1.84) | 0.92(0.75-1.13) |
| **Ever use cannabis/ amphetamine** (Yes vs No) | 0 .74(0.39-1.39) | 1.13(0.78-1.66) | 0.58(0.28-1.20) | 0.73(0.48-1.13) | 0.77(0.34-1.75) | 0.86(0.57-1.31) |
| **Two or more sexual partners** (yes vs. no) | 1.29(0.85-1.97) | **1.29(1.07-1.56)** | 0.74(0.47-1.17) | 1.04(0.84-1.28) | 0.82(0.53-1.28) | 1.12(0.93-1.35) |
| **Psychological distress items**: 0 1  2-5 | 1.17(0.62-2.21) | 0.96(0.73-1.25) | 1.71(0.87-3.35) | 1.01(0.78-1.32) | 1.37(0.71-2.63) | 0.90(0.67-1.20) |
|  | 0 .89(0.48-1.65) | 1.17(0.85-1.61) | 1.36(0.70-2.64) | 1.22(0.89-1.65) | 1.14(0.57-2.30) | 1.31(0.94-1.83) |
|  | 1 | 1 | 1 | 1 | 1 | 1 |
| **School attendance** (no vs. yes) | 0 .79(0.52-1.21) | 0.92(0.71-1.18) | 0.97(0.63-1.49) | 1.01(0.82-1.23) | 0.91(0.56-1.47) | 0.89(0.73-1.09) |
| **Peer support** (No vs Yes) | 1.26(0.84-1.90) | 1.12(0.90-1.40) | 1.07(0.70-1.63) | 1.07(0.85-1.34) | 0.96(0.57-1.61) | 1.12(0.90-1.39) |
| **Parental support**: Low  Medium  High | 1.64(0.97-2.78) | **1.52(1.13-2.04)** | **1.83(1.16-2.88)** | **1.60(1.24-2.05)** | **2.68(1.63-4.41)** | **1.84(1.36-2.48)** |
|  | 1.16(0.61-2.20) | 1.08(0.83-1.39) | 1.24(0.71-2.18) | 1.13(0.91-1.40) | 1.46(0.77-2.74) | 1.11(0.81-1.54) |
|  | 1 | 1 | 1 | 1 | 1 | 1 |
